# All subtypes of the Pmp adhesin family are implicated in chlamydial virulence and show species-specific function

**DOI:** 10.1002/mbo3.186

**Published:** 2014-07-01

**Authors:** Elisabeth Becker, Johannes H Hegemann

**Affiliations:** Funktionelle Genomforschung der Mikroorganismen, Heinrich-Heine Universität DüsseldorfDüsseldorf, Germany

**Keywords:** *Chlamydia pneumoniae*, *Chlamydia trachomatis*, Pathogen, Adhesin, Pmp protein family, Virulence

## Abstract

The bacterial pathogens *Chlamydia trachomatis* and *C. pneumoniae* are obligate intracellular parasites, cause a number of serious diseases, and can infect various cell types in humans. Chlamydial infections are probably initiated by binding of the bacterial outer membrane protein OmcB to host cell glycosaminoglycans (GAGs). Here, we show that all nine members of the polymorphic membrane protein (Pmp) family of *C. trachomatis* mediate adhesion to human epithelial and endothelial cells. Importantly, exposure of infectious particles to soluble recombinant Pmps blocks subsequent infection, thus implicating an important function of the entire protein family in the infection process. Analogous experiments with pairs of recombinant Pmps or a combination of Pmp and OmcB revealed that all Pmps probably act in an adhesion pathway that is distinct from the OmcB-GAG pathway. Finally, we provide evidence that the Pmps of *C. trachomatis* and *C. pneumoniae* exhibit species and tissue specificity. These findings argue for the involvement of *C. trachomatis* Pmps in the initial phase of infection and suggest that they may interact with a receptor other than the epidermal growth factor receptor recently identified for their counterparts in *C. pneumoniae*.

## Introduction

*Chlamydiae* are obligate intracellular bacteria that are responsible for a wide range of diseases of significant importance to public health. These pathogens are characterized by a unique biphasic life cycle consisting of two developmental forms, the infectious but quiescent extracellular elementary body (EB) and the metabolically active reticulate body (RB), which replicates exclusively within an intracellular vacuole, called inclusion (Moulder 1991). Despite the common developmental cycle, *Chlamydia* species display a high degree of diversity in host range, tissue tropism, and disease outcomes.

*Chlamydia trachomatis* is the major cause of trachoma leading to blindness by scarring of the cornea (serovars A–C), and of sexually transmitted diseases including urethritis, cervicitis, and salpingitis (serovars D–K). Untreated infections by these urogenital pathogens can lead to infertility in women and increase the risk of ectopic pregnancy (Schachter 1999). The lymphogranuloma venereum (LGV) biovars L1–L3 not only cause urogenital diseases but can also infiltrate local lymph nodes, which ultimately results in systemic infection. *Chlamydia pneumoniae* is a prevalent cause of community-acquired pneumonia, bronchitis, and pharyngitis and is also implicated in chronic diseases such as atherosclerosis (Grayston 2000).

Attachment to, and invasion of, cells are key steps in chlamydial development and pathogenesis, because blockage of these processes can inhibit subsequent infection (summarized in Hegemann and Moelleken 2012). Exposure of the infectious particles to heat or trypsin alters their adherence characteristics, which suggests that proteins or parts of proteins function as chlamydial adhesins (Vretou et al. 1989). In subsequent studies, several chlamydial proteins have been linked to the adhesion process. These include the major outer membrane protein of the *C. muridarum* strain that causes pneumonia in mice (Su et al. 1996), heat shock protein 70 from *C. trachomatis* (Raulston et al. 2002), and OmcB from both *C. trachomatis* and *C. pneumoniae* (Stephens and Lammel 2001). Further work identified the chlamydial outer membrane protein OmcB as an adhesin that binds to heparan sulfate-like glycosaminoglycans (GAGs) on the surface of human target cells, which is probably involved in the initial attachment of EBs to the host cell surface (Zhang and Stephens 1992; Fechtner et al. 2013). Interestingly, the GAG specificity of *C. trachomatis* OmcB reflects biovar-specific differences which might account, at least in part, for tissue tropism and the spread of the pathogen (Moelleken and Hegemann 2008; Fechtner et al. 2013). However, blocking of the OmcB-GAG interaction by various means always inhibited infection by no more than 90%, a finding which points to the involvement of additional chlamydial adhesin-receptor interactions (Zhang and Stephens 1992; Wuppermann et al. 2001; Fadel and Eley 2007; Moelleken and Hegemann 2008).

Immunoblotting experiments have identified several Pmps located in the chlamydial outer membrane complex (COMC) of *C. abortus* and *C. pneumoniae* as immunodominant antigens in infected hosts (Longbottom et al. 1996, 1998; Knudsen et al. 1999). Bioinformatic analysis of the genome sequences then revealed the full extent of this novel gene family, which comprises nine members in *C. trachomatis* (*pmpA* through *pmpI*) and 21 members in *C. pneumoniae* (Grimwood and Stephens 1999; Kalman et al. 1999). The *C. trachomatis pmp* gene family has been subdivided on phylogenetic grounds into the six subtypes: *A* (*pmpA*), *B* (*pmpB* and *pmpC*), *D* (*pmpD*), *E* (*pmpE* and *pmpF*), *G* (*pmpG* and *pmpI*), and *H* (*pmpH*) (Grimwood and Stephens 1999). The larger *C. pneumoniae* CWL029 *pmp* family (*pmp1* to *pmp21*) also comprises representatives of all these subtypes, but is characterized by a striking expansion of subtype *G*, which includes 13 members (*pmp1* through *pmp13*), and subtype E with four genes (*pmp15* through *pmp18*), while other subtypes are represented by a single gene each (Grimwood and Stephens 1999; Kalman et al. 1999). However, five of the genes among the expanded subtypes *E* and *G* (*pmp3* through *pmp5*, *pmp12* and *pmp17*) probably do not code for functional products (Grimwood et al. 2001).

The *pmp* genes represent remarkable 13.6% and 17.5% of the chlamydia-specific coding capacity in *C. trachomatis* and *C. pneumoniae*, respectively (Rockey et al. 2000). The relative abundance of the *pmp* genes within the highly reduced chlamydial genome, and the presence of the Pmp family in numerous *Chlamydia* species imply that Pmps play an essential role in chlamydial biology (Grimwood and Stephens 1999; Read et al. 2000, 2003; Thomson et al. 2005).

All Pmps are characterized by the presence of multiple repeats of GGA (I, L, V) and FxxN tetrapeptide motifs within the N-terminal half of the proteins and by a typical autotransporter structure, with a N-terminal Sec-dependent leader sequence, followed by a passenger domain and a C-terminal *β*-barrel (Grimwood and Stephens 1999; Henderson and Lam 2001). Like other autotransporter proteins, many Pmps undergo complex proteolytic processing (Vandahl et al. 2002; Wehrl et al. 2004; Kiselev et al. 2009; Swanson et al. 2009; Saka et al. 2011). Several of the *C. pneumoniae* and all *C. trachomatis* Pmps have been shown to be located on the chlamydial surface (Montigiani et al. 2002; Vandahl et al. 2002; Wehrl et al. 2004; Crane et al. 2006; Kiselev et al. 2009; Swanson et al. 2009; Moelleken et al. 2010; Tan et al. 2010). Interestingly, the Pmps of *C. trachomatis* and *C. abortus* exhibit variable expression in cell culture and variable Pmp-specific antibody profiles have been detected in *C. trachomatis*-infected patient sera (Tan et al. 2009, 2010; Carrasco et al. 2011; Wheelhouse et al. 2012). However, the possible role(s) of Pmps and the mechanisms that drive their diversification remained elusive.

An early clue to the function of the Pmps came from neutralization studies which showed that antibodies directed against Pmp2, Pmp10, Pmp21, and PmpD blocked subsequent infection by *C. pneumoniae* and *C. trachomatis*, respectively, pointing to a role for Pmp2, Pmp10, Pmp21, and PmpD in the initial phase of infection (Wehrl et al. 2004; Finco et al. 2005; Crane et al. 2006; Moelleken et al. 2010). Subsequent detailed studies of the *C. pneumoniae* proteins Pmp6, Pmp20, and Pmp21, which represent three different Pmp subtypes, ascertained that they act as adhesins and mediate attachment of chlamydial EBs to human epithelial cells (Moelleken et al. 2010). Interestingly, multiple domains of Pmp21 displayed strong adhesion properties, and subsequent mutational experiments revealed the two repetitive peptide motifs GGA (I, L, V) and FxxN to be essential for the adhesion function. Importantly, pretreatment of human cells with recombinant Pmp6, Pmp20, or Pmp21 reduced EB attachment and subsequent infectivity providing direct evidence for the critical role of the three Pmps in *C. pneumoniae* pathogenesis. More recently, the epidermal growth factor receptor (EGFR) was identified as the receptor for the *C. pneumoniae* adhesin Pmp21 (Molleken et al. 2013).

Interestingly, members of the Pmp families show large species- and subtype-specific sequence heterogeneity. Proteins within each Pmp subclass show significant sequence identity to each other. For example, in *C. trachomatis* serovar E, the two members of subtype G (PmpG, PmpI) show 25% overall identity, and PmpB and PmpC from subtype B are 43% identical to each other. Indeed, the subtypes have maintained a significant protein sequence similarity even across species. Between *C. trachomatis* E and *C. pneumoniae* CWL029, Pmp identities range from 23% to 32% for the PmpG subtype members and 33% for the PmpD subtype members PmpD and Pmp21, indicating some level of functional similarity across chlamydial species. In contrast, in *C. trachomatis* E, identity levels between proteins of the different subtypes are low, mostly between 13% and 19% (Grimwood and Stephens 1999).

In this study we show, for the first time and by means of two different experimental approaches, that all nine members of the Pmp family of *C. trachomatis* serovar E can adhere to human cells. Moreover, *C. trachomatis* and *C. pneumoniae* Pmps reveal differential binding capabilities depending on the human cell-type analyzed. Importantly, all nine recombinant Pmps can neutralize a *C. trachomatis* infection suggesting an important role for all of the Pmp adhesins in the infection process. Furthermore, our combination experiments using mixtures of two different recombinant Pmps suggest that they act in the same pathway, while the results obtained for a Pmp in combination with OmcB are indicative of two separate pathways. Finally, pretreatment of human epithelial cells with recombinant Pmps inhibits chlamydial infection in a species-specific manner.

## Experimental Procedures

### Reagents and antibodies

Gastrografin was purchased from Schering. *α*-Mannosidase, Cy3-conjugated anti-mouse antibodies, poly-l-lysine, protein-coated latex beads (diameter 1 *μ*m, green fluorescent), and cell dissociation solution were obtained from Sigma (St. Louis, Missouri, USA). The anti-His5 antibody was procured from Qiagen (Hilden, Germany), the anti-His6 antibody from Roche (Basel, Switzerland), the anti-V5 antibody from Invitrogen (Carlsbad, California, USA), the AP-conjugated anti-mouse antibody from Promega (Madison, Wisconsin, USA), and the monoclonal Fluorescein isothiocyanate (FITC)-conjugated antibody against chlamydial Lipopolysaccharide (LPS) from Bio-Rad (Hercules, California, USA).

### Bacterial strains, yeast strains, and cell culture

*Escherichia coli* strain XL-1 blue (Stratagene, La Jolla, California, USA) was used for protein expression and plasmid amplification. *Chlamydia pneumoniae* GiD and *C. trachomatis* serovar E (DK-20; Institute of Ophthalmology, London, UK) were propagated in HEp-2 cells as described (Jantos et al. 1997). Chlamydial EBs were purified using 30% gastrografin. The *Saccharomyces cerevisiae* strain CEN.PK2 was used for homologous recombination. The strain used for adhesion experiments (MATa *ura*3-52 *trp*1 *leu*2Δ1::pCM149(*LEU2*) *his*3Δ200 *pep4:HIS3::loxP-KanMx-loxP-Met25p-GFP prb*1Δ.6R*can*1 *tetO7 AGA1*::pIU211(*URA3*)) was derived from strain YKM2 (Invitrogen), and constructed by Dr. K. Mölleken. HEp-2 (ATCC: CCL-23) and HeLa229 cells (ATCC: CCL-2.1) were cultured in Dulbecco's Modified Eagle's Medium (DMEM) medium; human umbilical vein endothelial cells (HUVEC), kindly provided by Prof. Dr. B. Homey, were cultivated through four passages in endothelial cell medium.

### DNA manipulations and plasmid construction

Plasmids containing chlamydial genes were generated by homologous recombination in *S. cerevisiae* as described (Moelleken and Hegemann 2008). Fragments of *pmp* genes and of *cpn0498* were amplified from genomic DNAs of *C. pneumoniae* GiD and *C. trachomatis* serovar E DK-20 by PCR (using the primer pairs listed in Table S1) and either cloned directly into the *EcoR*I/*Not*I site of the yeast expression vector pYD1 (Invitrogen) or into the *Sma*I site of the *E. coli* expression vector pKM32 (Moelleken et al. 2010) or into the *Sma*I site of the *E. coli* expression vector pFT8 (T. Fechtner, unpubl. data). All constructs were sequenced prior to use.

### Protein expression and affinity purification of His6-tagged proteins

Expression in *E. coli* and purification of N-terminally His6-tagged fusion proteins was performed under denaturing conditions following the manufacturer's instructions (Qiagen). Proteins were renatured by dialysis against Phosphate Buffered Saline (PBS) and analyzed after sodium dodecyl sulfate polyacrylamide gel electrophoresis (SDS-PAGE) with an anti-His6 antibody.

### Immunoblot analysis

SDS-PAGE and immunoblot analysis were performed as described (Sambrook et al. 1989). The His6-tagged recombinant proteins purified from *E. coli* were detected with a monoclonal anti-His6 antibody and visualized with AP-conjugated anti-mouse antibody. The His_6_-tagged Aga2 fusion proteins from *S. cerevisiae* were detected with a monoclonal anti-His_5_ antibody and visualized with an AP-conjugated anti-mouse antibody.

### Immunofluorescence microscopy

Immunofluorescence microscopy of yeast cells expressing Aga2p or Aga2p fusion proteins was performed on samples of 5 × 10^6^ cells grown for 24 h under inducing conditions. Cells were washed twice with PBS and fixed on glass slides coated with poly-l-lysine. Slides were incubated with anti-V5 and Cy3-conjugated anti-mouse antibodies.

### Yeast adhesion assays

For expression of Aga2p or Aga2p fusion proteins in strain YKM2, cells were grown in accordance with the manual supplied by Invitrogen. Yeast adhesion assays were performed with HEp-2 or HeLa cells cultivated on glass coverslips as described previously (Moelleken and Hegemann 2008). To visualize adherent cells, expression of endogenous Green Fluorescent Protein (GFP) was induced by growing the cells in selective synthetic dextrose (SD) medium without methionine. The number of fluorescent yeast cells attached to 10^3^ HEp-2 or HeLa cells were counted under the microscope. Each experiment was repeated three times.

### Adhesion assays with protein-coated latex beads

Adhesion assays with protein-coated latex beads were performed as described (Dersch and Isberg 1999). Coating efficiency was estimated by immunoblotting prior to use. Confluent monolayers of HEp-2, HeLa, or HUVE cells were grown in 24-well plates and incubated with a 10-fold excess of protein-coated latex beads (diameter 1 *μ*m, green fluorescent) for 1 h at 37°C. Cells were washed twice with PBS, detached with cell dissociation solution, fixed with 3% formaldehyde for 20 min at RT, and analyzed by flow cytometry using a FACSAria instrument (BD Biosciences, San Jose, California, USA).

### Infection inhibition assays

These assays were performed as previously described (Moelleken and Hegemann 2008). Briefly, HEp-2 cells were grown on glass coverslips (12-mm diameter) for 48 h and incubated (as confluent monolayers) with 250 *μ*L of medium containing recombinant protein (12.5–400 *μ*g mL^−1^ in PBS) for 2 h at 37°C. Purified chlamydial Elementary Bodies (EBs) (multiplicity of infection (moi) 20) were added to the protein suspension and incubated for 2 h at 37°C without centrifugation in order to avoid any influence of the centrifugation procedure on the adhesion/infection process (Moelleken and Hegemann 2008). The cells were then washed three times, covered with chlamydial growth medium, incubated for 24 h (*C. trachomatis* infection) or 48 h (*C. pneumoniae* infection) at 37°C, and fixed with 96% methanol for 2 min. Each monolayer was then washed three times with PBS. For detection of chlamydial inclusions, a monoclonal FITC-conjugated antibody directed against chlamydial LPS was used. Cells were viewed using a Zeiss Axioskop. Typically the number of inclusions in the untreated control experiments was between 500 and 600 per 10^3^ human cells. Inclusion numbers were determined and expressed as a percentage of the number found in PBS-treated samples.

## Results

### All nine *C. trachomatis* Pmps are capable of mediating attachment to human cells

**Figure 1:**
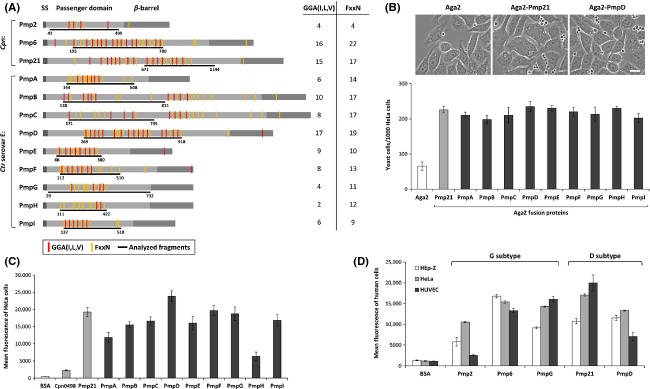
The *Chlamydia trachomatis* Pmps mediate adhesion to human cells. (A) Schematic representation of Pmp2, Pmp6, and Pmp21 from *C. pneumoniae* and of all nine Pmps from *C. trachomatis* serovar E. The characteristic three-domain structure of Pmps, with the N-terminal signal sequence (SS), the passenger domain, and the C-terminal *β*-barrel domain are indicated by gray boxes. The positions of the amino acid motifs GGA (I, L, V) and FxxN are indicated by red and yellow lines and the total number of each motif type found is given on the right. The location and extent of the Pmp protein fragments studied here are indicated by the black underlines and amino acid positions. (B) Top: Representative micrographs of HeLa cells after incubation with yeast cells expressing the indicated proteins (A) on their surface. Bar: 10 *μ*m. Bottom: Quantification of number of Pmp-presenting yeast cells bound to 10^3^ HeLa cells. Negative control: yeast cells expressing Aga2. Positive control: yeast cells expressing Aga2-Pmp21. (C) Binding of latex beads coated with the indicated recombinant proteins to HeLa cells. Fluorescent latex beads (1 × 10^6^), coated with 200 *μ*g mL^−1^ of the indicated proteins, were incubated, in 10-fold excess, with 10^5^ HeLa cells. The mean fluorescence of 10^4^ HeLa cells with bound beads was then determined by flow cytometry. (D) Binding of latex beads coated with selected *C. pneumoniae* and *C. trachomatis* Pmps of the D and G subtypes to epithelial (HEp-2, HeLa) and endothelial (HUVE) cells. In this assay, the three different cell types were incubated in parallel with the same batch of coated beads, thus excluding potential differences in coating efficiency. Results in B–D are derived from three independent experiments (*n* = 3). Data shown are means ± standard deviations.

In previous studies, Pmp6, Pmp20, and Pmp21 from *C. pneumoniae* were characterized as bacterial adhesins that are important for the infection of human epithelial cells (Moelleken et al. 2010). In order to elucidate the function of the Pmp family of *C. trachomatis*, all nine Pmps from the urogenital-tract pathogen *C. trachomatis* serovar E, strain DK-20, were analyzed for their adhesion properties using the heterologous yeast display system (Moelleken and Hegemann 2008). As the motifs GGA (I, L, V) and FxxN that are characteristic of Pmps had been found to be essential for adhesion mediated by Pmp21 from *C. pneumoniae* (Moelleken et al. 2010), we focused our attention on regions within the passenger domains of all nine *C. trachomatis* Pmps that displayed these motifs in significant numbers (Fig.1A). The selected passenger domain fragment of each *C. trachomatis* Pmp was fused to the yeast surface protein Aga2, and yeast cells bearing the Aga2-Pmp fusion proteins on their surfaces were analyzed for binding to human epithelial HeLa cells (Fig.1A and B). All Aga2-Pmp fusion proteins were expressed in approximately similar amounts and could be detected on the yeast cell surface (Fig. S1). Yeast cells presenting Aga2 alone showed only a weak binding to human cells (70 yeast cells/10^3^ HeLa cells). In contrast, yeast cells expressing Aga2 fused to Pmp21 of *C. pneumoniae* (positive control) exhibited more than threefold higher adhesion to human cells (Fig.1B). Most strikingly, yeast cells expressing any one of the nine *C. trachomatis* Pmps adhered with comparable affinity to HeLa cells in this assay (200–230 yeast cells/10^3^ HeLa cells) (Fig.1B).

In order to confirm these findings, we performed bead-based assays with recombinant Pmp passenger-domain fragments (see Fig.1A) expressed in and purified from *E. coli* (Dersch and Isberg 1999). The different Pmp variants depicted in Figure1A were coupled as recombinant His6-tagged proteins to green fluorescent latex beads. The coating efficiency estimated by immunoblotting revealed only slight differences in the amounts of each protein coupled to the beads (Fig. S2). The positive control, Pmp21-coated beads, showed strong binding to HeLa cells (mean fluorescence ∼20,000) in comparison to a very low binding of BSA coated beads (mean fluorescence ∼500) (Fig.1C). Moreover, an additional hypothetical chlamydial protein, CPn0498, originally identified as being EB-surface located (Montigiani et al. 2002), was tested, and only weak adhesion could be detected (mean fluorescence ∼2000) (Fig.1C). Again, all *C. trachomatis* Pmps exhibited significant adhesion to HeLa cells (Fig.1C). In contrast to the results from the yeast display assay (Fig.1B), latex beads coated with recombinant *C. trachomatis* Pmps revealed distinct binding affinities for HeLa cells (Fig.1C). For example, the affinity of PmpD (mean fluorescence ∼25,000) was about four times higher than that of PmpH (∼6000). The other seven Pmps, meaning the majority of *C. trachomatis* Pmps, displayed intermediate attachment efficiency to epithelial cells (mean fluorescence ∼12,000–20,000). Hence, both the yeast surface display and the bead assay revealed that all nine members of the *C. trachomatis* Pmp family exhibit significant adhesion to human epithelial cells.

### Pmps show differential binding to epithelial and endothelial cells

Since it has been speculated that the Pmps of *C. trachomatis* may have a niche-specific function in bacterial pathogenesis (Li et al. 2005; Gomes et al. 2006; Nunes et al. 2007; Tan et al. 2010), we wondered whether the Pmps would exhibit similar adhesion capacities to different epithelial and endothelial cell types. For this study, we focused on Pmp2, Pmp6, and Pmp21 from *C. pneumoniae* and on PmpG and PmpD from *C. trachomatis* as representatives of the two Pmp subtypes G (2, 6, and G) and D (21 and D), respectively. Latex beads coated with each of the recombinant Pmps were incubated with one endothelial (primary HUVE) and two different epithelial (HEp-2, HeLa) cell types (Fig.1D). All Pmps analyzed exhibited significant but quantitatively distinguishable adhesion capacities for all three cell lines, in comparison to the BSA control. Overall, Pmp2 showed weaker adhesion than either of the other representatives of subtype G. In particular, the affinity of Pmp2 for HUVE cells was markedly lower (mean fluorescence ∼3000) than that of the other two subtype G candidates (Pmp6 ∼13,000; PmpG ∼16,000). Moreover, while Pmp6 showed comparable levels of adhesion to all three cell lines, Pmp2 and PmpG each exhibited a wider range of binding affinities for the different test cells. Significant binding differences were also observed for Pmp21 and PmpD, both of which belong to subclass D. Pmp21 showed the strongest binding to HUVE cells (mean fluorescence ∼20,000), while PmpD adhered most effectively to HeLa cells (mean fluorescence ∼13,000). Thus, our findings imply that each of the Pmps studied here displays a characteristic adhesion profile for the epithelial and endothelial cell lines tested.

### All Pmp adhesins are important for the *C. trachomatis* infection

**Figure 2:**
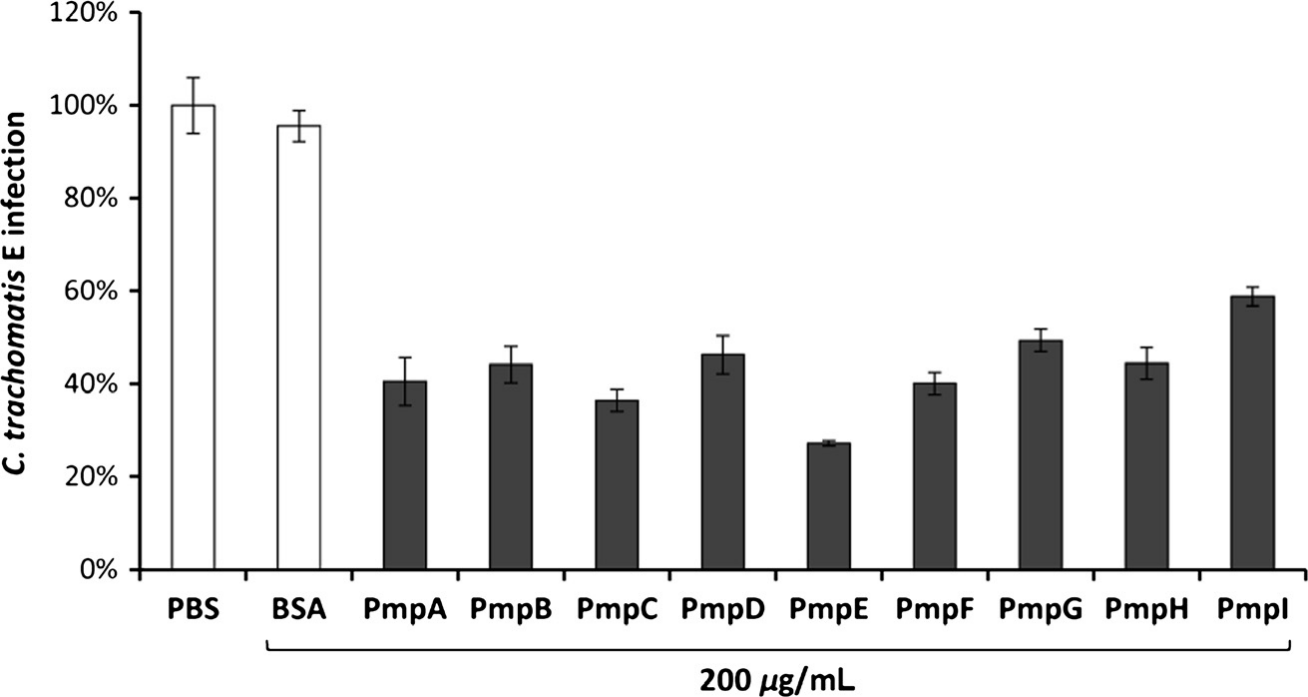
Purified recombinant forms of all Pmp adhesins can all interfere with the *Chlamydia trachomatis* infection process. HEp-2 cells (1 × 10^6^) were incubated with PBS, BSA (200 *μ*g mL^−1^), or the indicated recombinant protein (at 200 *μ*g mL^−1^) prior to exposure to purified *C. trachomatis* EBs (moi 20). Cells were fixed at 24 h postinfection and the number of inclusions formed was determined by microscopy. The number of inclusions per 10^3^ human cells was determined, and is expressed as a percentage of the number found in PBS-treated samples. Results are derived from three independent experiments (*n* = 3). Data shown are means ± standard deviations.

Our data strongly suggest that all nine *C. trachomatis* Pmps are capable of binding to human cell lines, and thus may be involved in mediating infections in humans. To test the relevance of the Pmps for infection more directly, we asked whether soluble recombinant Pmps could interfere with the establishment of a *C. trachomatis* infection. For this purpose, we preincubated HEp-2 cells with equal amounts of each of the recombinant Pmps. All nine *C. trachomatis* Pmps were found to inhibit infection upon subsequent exposure to *C. trachomatis* EBs (Fig.2). The degree of inhibition ranged from 40% to 70% in comparison to PBS- and BSA-treated cells. Pretreatment with recombinant PmpE had the strongest inhibitory effect with ∼70% infection inhibition. Furthermore, the majority of the *C. trachomatis* Pmps reduced infectivity by 50–60%, and can be ranked in order of decreasing efficacy as follows PmpC, PmpF, PmpA, PmpB, PmpH, PmpD, and PmpG. The least effective of the set was PmpI, which inhibited infectivity by ∼40%. Taken together, these results demonstrate that preincubation with recombinant *C. trachomatis* Pmps inhibits the cognate chlamydial infection. Thus, all Pmps from *C. trachomatis* can interact with human cells and apparently participate in the infection process.

### Soluble PmpD and PmpG redundantly inhibit infection by *C. trachomatis*

**Figure 3:**
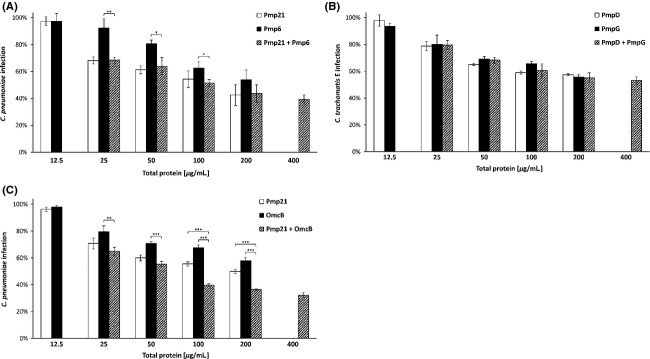
*Chlamydia trachomatis* PmpD and PmpG bind to the same host cell target. HEp-2 cells were preincubated with increasing amounts of either a single recombinant protein (12.5–200 *μ*g mL^−1^) or of a one-to-one mixture of two proteins (25–400 *μ*g mL^−1^) prior to exposure to cognate *Chlamydia* EBs. For a given total protein concentration, each bar represents treatment with the same amount of protein. For example, in B, total protein 50 *μ*g mL^−1^ indicates preincubation with 50 *μ*g mL^−1^ PmpD alone (open bar), 50 *μ*g mL^−1^ PmpG alone (black bar) or a one-to-one mixture of 25 *μ*g mL^−1^ PmpD and 25 *μ*g mL^−1^ PmpG, that is, again 50 *μ*g mL^−1^ in total (hatched bar). The assay was performed as described in the legend to Figure2. Results are derived from three independent experiments (*n* = 3). Data shown are means ± standard deviations. (A) HEp-2 cells were preincubated with recombinant Pmp21 (the PmpD ortholog) and/or Pmp6 (the PmpG ortholog) prior to infection with *C. pneumoniae*. (B) HEp-2 cells were preincubated with recombinant PmpD and/or PmpG prior to infection with *C. trachomatis*. (C) HEp-2 cells were preincubated with recombinant Pmp21 and/or full-length OmcB prior to infection with *C. pneumoniae*. Only statistically significant differences are denoted: *P < 0.05; **P < 0.01; ***P < 0.001.

The observation that all *C. trachomatis* serovar E Pmps may contribute to EB adhesion and, in soluble form, significantly interfere with the process of infection by *C. trachomatis*, suggests that they share a common mode of action. In order to explore whether different *C. trachomatis* Pmps have overlapping functions, two different recombinant Pmps belonging to distinct subtypes were utilized either singly or in combination for infection inhibition assays. Human cells were incubated with different concentrations of either recombinant Pmp6 or Pmp21 alone, or with a combination of both, prior to exposure to *C. pneumoniae* EBs (Fig.3A). Likewise, we preincubated human cells with recombinant PmpG and PmpD, separately or together, before adding *C. trachomatis* (Fig.3B). The recombinant *C. pneumoniae* Pmps, alone or in combination, inhibited subsequent infection in a dose-dependent manner, with PmpD at lower protein concentrations of 25 and 50 *μ*g mL^−1^ doing so somewhat more strongly than Pmp6 (Fig.3A). At a concentration of 200 *μ*g mL^−1^, the *C. pneumoniae* infection was inhibited by 55% with recombinant Pmp21 alone and by 45% with Pmp6 alone. Interestingly, preincubation of HEp-2 cells with both proteins together inhibited the infection by up to 55% when each was present at a concentration of 100 *μ*g mL^−1^ = 200 *μ*g mL^−1^ in total. Notably, this is identical to the level of inhibition measured for Pmp21 alone at 200 *μ*g mL^−1^ (Fig.3A). Importantly, at all total protein concentrations tested, the combination of both proteins (hatched bars) reduced subsequent infection no more strongly than the more effective of the two on its own (white or black bar). Thus, the efficacy of inhibition mainly depends on the total amount of Pmp protein used in the experiment, irrespective of whether a single Pmp protein or a combination of two different Pmp proteins is present. This was observed at all other concentrations studied here, and indicates that Pmp21 and Pmp6 have partially redundant effects in this infection inhibition assay, thus verifying and extending earlier observations for these two proteins (Moelleken et al. 2010). Preincubation of infectious *C. trachomatis* serovar E EBs with either recombinant PmpD or PmpG alone reduced subsequent infection by up to 45% at the maximum concentration tested (200 *μ*g mL^−1^). Interestingly, the one-to-one combination of both to give a total protein concentration of 200 *μ*g mL^−1^ inhibited infection to the same degree. Remarkably, the extent of the infection inhibition obtained for PmpD or PmpG alone or both proteins together was identical at all protein concentrations tested, strongly suggesting redundancy of PmpD and PmpG function (Fig.3B).

In order to determine whether OmcB acts in an adhesion pathway distinct from the Pmp pathway, we combined recombinant Pmp21 with recombinant OmcB in a *C. pneumoniae* infection inhibition experiment (Fig.3C). Blocking the binding sites on the host cell with recombinant OmcB protein dose-dependently reduced the *C. pneumoniae* infection by up to 40%, while Pmp21 inhibited by up to 50% (both at 200 *μ*g mL^−1^). In contrast, pretreatment of host cells with equal amounts (100 *μ*g mL^−1^ each) of OmcB and Pmp21 (protein concentration in total 200 *μ*g mL^−1^) reduced the subsequent infection by 65%. This additive effect of the two proteins was observed at all protein concentrations tested (Fig.3C). Since the combination of recombinant Pmp21 and OmcB consistently inhibited subsequent infection inhibition more than did either protein alone, we conclude that Pmp21 and OmcB function in two different adhesion pathways.

In summary, these data indicate that Pmp21 and Pmp6 recognize at least partially overlapping structures on the surface of the target cell during chlamydial adhesion, as do PmpD and PmpG likewise. This suggests that these Pmps contribute to one infection pathway. Furthermore, our data suggest that OmcB and Pmp21 additively inhibit infection, indicating that they interact with different host cell targets during chlamydial infections.

### Pmps inhibit chlamydial infection in a species-specific manner

**Figure 4:**
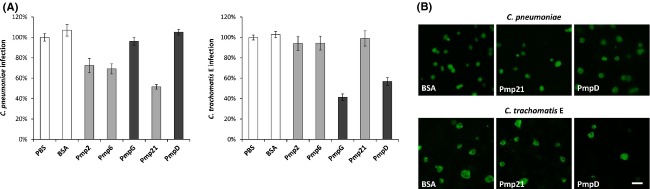
Recombinant Pmps from *Chlamydia trachomatis* do not inhibit infection by *C. pneumoniae* and vice versa. (A) HEp-2 cells were incubated with PBS, BSA (200 *μ*g mL^−1^), or the indicated recombinant protein (at 200 *μ*g mL^−1^) prior to infection with *C. pneumoniae* (left panel) or *C. trachomatis* (right panel) EBs. Cells were fixed 48 h (*Cpn*) or 24 h (*Ctr*) after infection. The assay was performed as described in the legend to Figure2. Results are derived from three independent experiments (*n* = 3). Data shown are means ± standard deviations. (B) Immunofluorescence microscopy of the experiment shown in (A). For detection of chlamydial inclusions, methanol-fixed cells were probed with an antibody directed against chlamydial LPS. Bar 10 *μ*m.

In previous studies, PmpD antiserum was shown to neutralize *C. trachomatis* serovars but failed to neutralize the infectivity of *C. muridarum*, identifying PmpD as a species- but not (necessarily) serovar-specific infection factor (Crane et al. 2006). Given the common function of *C. pneumoniae* and *C. trachomatis* Pmps as adhesins, we examined if recombinant *C. trachomatis* Pmps could reduce infectivity of *C. pneumoniae* and vice versa (Fig.4A and B). Again, Pmp2, Pmp6, and Pmp21 of *C. pneumoniae* and PmpG and PmpD of *C. trachomatis* were selected as representatives of the Pmp subtypes G and D. Preincubation of human cells with equal amounts of recombinant *C. pneumoniae* Pmps (200 *μ*g mL^−1^) significantly inhibited subsequent infection by *C. pneumoniae*. Recombinant Pmp2 and Pmp6 each reduced infectivity by 30%, while Pmp21 led to a 50% reduction in infection (Fig.4A left, Fig.4B top panel). However, *C. trachomatis* PmpG and PmpD was no more effective in blocking infection by *C. pneumoniae* infection than the PBS or BSA control (Fig.4A, left panel).

In the converse experiment, *C. trachomatis* serovar E infectivity was reduced by 60% and 40% respectively, when cells were pretreated with soluble PmpG or PmpD. Again, the *C. trachomatis* infection was not affected when the human cells were preincubated with *C. pneumoniae* Pmp2, Pmp6, or Pmp21 (Fig.4A right, Fig.4B bottom panel). Thus, our findings imply that recombinant Pmps inhibit chlamydial infection in a species-specific manner, indicating differences in adhesion mechanism between *C. pneumoniae* and *C. trachomatis* Pmps.

## Discussion

For obligate intracellular pathogens, such as members of the genus *Chlamydia*, adhesion to and subsequent internalization by the host cell are two critical steps in the early phase of the infection cycle. Numerous studies have led to the assumption that chlamydial pathogens utilize multiple mechanisms for host cell invasion, which are likely to vary with the target cell type and the chlamydial species involved (Hegemann and Moelleken 2012). In most chlamydial infections, the initiate adhesion process begins when the bacterial outer membrane protein OmcB interacts with heparan sulfate-like GAGs on the host cell. Blockage of this attachment pathway significantly reduces, but does not completely abrogate infection, indicating that additional mechanisms come into play here. The identification of the polymorphic membrane protein PmpD of *C. trachomatis* as the target of neutralizing antibodies that reduce the infectivity of all serovars tested pointed to a role of this protein in early aspects of infection by *C. trachomatis* (Crane et al. 2006). Indeed, the present study shows that all nine members of the Pmp family encoded by serovar E of *C. trachomatis*, a urogenital pathogen, can mediate adhesion to epithelial and endothelial human cells in vitro, and that they are important for infection. Moreover, we provide evidence that recombinant Pmp2 from *C. pneumoniae* exhibits adhesive capacities to epithelial and endothelial cells, which is in agreement with other studies showing that specific anti-Pmp2 antibodies neutralize a *C. pneumoniae* infection (Finco et al. 2005).

Pmps, including those of *C. trachomatis*, can be subdivided on phylogenetic grounds into six subtypes, and exhibit broad interspecies, as well as inter- and intrasubtype sequence diversity, with protein sequence identities ranging from 15% to 40% (Grimwood and Stephens 1999). Interestingly however, despite this sequence heterogeneity all nine *C. trachomatis* Pmps showed significant adhesion activity in two very different assays. Surface display of individual Pmps on yeast cells conferred very similar adhesion capacities for human cells on the different carrier strains, while the use of recombinant Pmps coupled to latex beads revealed some significant quantitative differences among them. These differences might reflect Pmp-specific refolding properties during recombinant production and/or differences in Pmp oligomerization. Indeed, PmpD oligomeric protein structures have been isolated from infectious EBs (Tanzer et al. 2001; Swanson et al. 2009). Thus, the adhesive capacity of individual Pmp protein-coated beads may in part depend on the particular Pmp oligomer formed by the recombinant protein fragment used. The fact that the same Pmp protein fragments show very comparable adhesion capacity when surface presented on yeast cells may thus indicate very similar adhesion capacities of all nine Pmp proteins.

Importantly, all nine recombinant Pmps were capable of blocking infection by *C. trachomatis* – a finding which directly implicates the whole family in the infection process. The adhesion function found for all Pmps is consistent with the fact that all family members are localized on the bacterial cell surface (Tan et al. 2010). However, protein localization on the EB cell surface does not necessarily imply a role in host cell adhesion, as the hypothetical chlamydial protein CPn0498, originally identified as being expressed in and located on EBs (Montigiani et al. 2002), showed very little adhesive capacity in the bead assay. The strong adhesive property common to all nine Pmp members, on one hand, and the sequence divergence within and between species and serovars on the other, suggest that all Pmps possess shared functional elements. The prime candidates lie in passenger domains of the Pmps, which are characterized by large numbers of short repetitive peptide motifs, GGA (I, L, V) and FxxN, which were shown to be essential for adhesion to human cells mediated by *C. pneumoniae* Pmp21. Moreover, a Pmp21-derived synthetic peptide harboring one GGAI and one FxxN motif attenuates a *C. pneumoniae* infection while a scrambled peptide had no effect, proving the importance of the tetrapeptide motifs for Pmp21 function (Moelleken et al. 2010). As structure predictions indicate that the passenger domains of all Pmps comprise *β*-helical structures (Bradley et al. 2001), it is conceivable that structural conservation within this domain contributes to their overlapping functions as adhesins.

In both assay systems, PmpD emerged as the strongest adhesin suggesting that PmpD might be of particular relevance to *C. trachomatis* infections. This is supported by the fact that PmpD is the second most highly conserved member of the Pmp family (Gomes et al. 2006). Moreover, it has been found that each member of the Pmp family exhibits a specific pattern of variability in expression, when *C. trachomatis* is propagated in cell culture (Tan et al. 2010). Interestingly, with OFF rates as low as 0.1–1%, PmpD was the only Pmp found to be expressed in almost all intracellular inclusions, which again argues for a crucial function of PmpD in the infection cycle. Moreover, since *C. trachomatis* Pmps are known to be targeted by the host's adaptive immune system, variable expression of the Pmps would extend antigenic diversity (Tan et al. 2010; Carrasco et al. 2011; Wheelhouse et al. 2012) and facilitate avoidance of immune surveillance. Accordingly, patients infected with *Chlamydia* show a diverse serological response to the various Pmps (Bunk et al. 2008; Tan et al. 2009). Thus, the variable expression profile of a group of nine Pmp adhesins with similar functionality would ensure that while the infectious chlamydial cells derived from different inclusions carry different, inclusion-specific subsets of Pmp adhesins to escape the adaptive immune system, preservation of the essential adhesive capacity of the infectious EBs would be guaranteed.

Our studies revealed that the Pmps exhibit a broad adhesion profile, binding to two different epithelial cell lines (HEp-2, HeLa) and to primary endothelial HUVE cells, as assessed by the bead assay. A detailed inspection of the data indicates that neither the different Pmps from one species nor the members of a single Pmp subtype displayed the same adhesion pattern, indicating that each Pmp harbors its individual adhesion profile. This argues that many members of the Pmp family may be important for niche adaptation (Tan et al. 2010; Carrasco et al. 2011). Indeed, in the past, Pmps have frequently been correlated with tissue tropism. For instance, *pmp* genes were found among a set of candidate ORFs which were associated with rectal tropism in serovar G isolates (Li et al. 2005). Polymorphism in Pmps has also been linked to adaptive evolution of cell-type/organ specificity of *C. trachomatis* serovars (Nunes et al. 2007). Therefore, Pmps seem to be under positive selection among different serovars, implying that they function in adaption to specific niches while likely keeping their adhesion capacities (Borges et al. 2012).

Prior exposure to purified recombinant versions of all nine *C. trachomatis* Pmps in soluble form can inhibit subsequent *C. trachomatis* infection, in agreement with published data for Pmp6, Pmp20 and Pmp21 of *C. pneumoniae* and PmpD of *C. trachomatis* (Wehrl et al. 2004; Crane et al. 2006; Moelleken et al. 2010). Interestingly, when recombinant PmpD and PmpG were used together in the infection blocking assay, no additive effect was observed. Likewise, preincubation of *C. pneumoniae* EBs with a combination of Pmp6 and Pmp21 blocked the infection to a level almost identical to that observed for either protein alone, in agreement with earlier data obtained for Pmp6 and Pmp20 of *C. pneumoniae* (Moelleken et al. 2010). These findings imply that the Pmps recognize the same host cell receptor or group of receptor molecules. Thus the recent identification of the human EGFR as a binding partner for the *C. pneumoniae* Pmp21 protein may indicate that Pmp6 and Pmp20 also recognize this receptor (Molleken et al. 2013). The question remains whether the different members of a chlamydial Pmp family bind as single molecules or, as seems more likely, as a multimeric complex on the bacterial cell surface. Indeed, homo- or hetero-oligomeric interactions have been predicted for Pmps of *C. trachomatis* and *C. psittaci* (Tanzer et al. 2001; Swanson et al. 2009). These interactions could be similar to the postulated multimerization of the *Haemophilus influenzae* Hap autotransporter, which is thought to be involved in cell–cell interaction (Cotter et al. 1995).

While the Pmps may act in the same adhesion pathway, our data show that blocking of OmcB binding sites with recombinant OmcB protein reduces infection by *C. pneumoniae* by up to 40%, while Pmp21 inhibited by ∼50%. Interestingly, both proteins together reduced the subsequent infection by 70% suggesting an additive effect (Fig.3C). Thus, we provide evidence here, for the first time, that the attachment of *C. pneumoniae* is a multistep process involving two distinct adhesion pathways, represented by an OmcB-GAG interaction and binding of the members of the Pmp family to separate receptor(s), perhaps EGFR, as has been shown for Pmp21. However, alternative models cannot be entirely ruled out at present. Since our studies indicate that *C. pneumoniae* and *C. trachomatis* Pmps act as adhesins, an analogous multistep attachment process is likely to hold for *C. trachomatis* EBs.

Despite the fact that the Pmps of *C. pneumoniae* and *C. trachomatis* have essentially similar functions, we have uncovered pivotal differences between the two species. Thus, the infection blocking assay revealed that *C. trachomatis* Pmps have little or no effect on the infectivity of *C. pneumoniae* and vice versa. Hence, recombinant Pmps inhibit chlamydial infection in a species-specific manner. This important finding is compatible with the hypothesis that, although *C. trachomatis* and *C. pneumoniae* share at least one attachment mechanism mediated by the OmcB-GAG interaction (Hegemann and Moelleken 2012), they differ in their respective requirements for host cell functions involved in the attachment and/or invasion process (Carabeo and Hackstadt 2001; Fudyk et al. 2002). Thus, the results of this study suggest that *C. trachomatis* PmpD, an ortholog of *C. pneumoniae* Pmp21, may not interact with EGFR. In future the identification of the interaction partner(s) for the *C. trachomatis* Pmp adhesin family will pave the way for a better understanding of the biological function of these proteins and provide insight into their species specificity. Such advances in Pmp biology will also uncover new targets for antichlamydial drugs and vaccines.

## Supporting Information

Figure S1 Yeast cells presenting chlamydial Pmps on their cell surface. (A) Detection of Aga2 and Aga2-Pmp fusion proteins on the yeast cell surface. Green fluorescent yeast cells expressing Aga2 and Aga2–Pmp fusion proteins were fixed and stained with an anti-V5 antibody (red) against the C-terminal V5-Tag. Top panel: Phase contrast microscopy. Bottom panel: Fluorescence microscopy. (B) Western blot analysis of Aga2 and Aga2-Pmp fusion proteins. Total protein extracts from yeast strains expressing Aga2 or Aga2–Pmp fusion proteins were digested with *α*-mannosidase to remove Aga2 *O*-glycosylation, resolved by SDS-PAGE and probed with an anti-His antibody. Protein size markers (in kDa) are indicated to the left of each blot.

Figure S2 Coating efficiency of latex beads. Western blot analysis of recombinant proteins coated on 2 × 107 latex beads (adhesion data shown in Fig. C). Proteins were removed from the bead surface by SDS-loading buffer and DTT, resolved by SDS-PAGE, and probed with an antibody against the N-terminal His tag. Lane M, molecular mass marker.

Table S1 List of oligonucleotides used for plasmid construction.
