# Supplementary figures and images for: All subtypes of the Pmp adhesin family are implicated in chlamydial virulence and show species-specific function
Source: Microbiologyopen. 2014 Jul 1;3(4):544–56. doi: 10.1002/mbo3.186 (PMC4287181; doi:10.1002/mbo3.186)

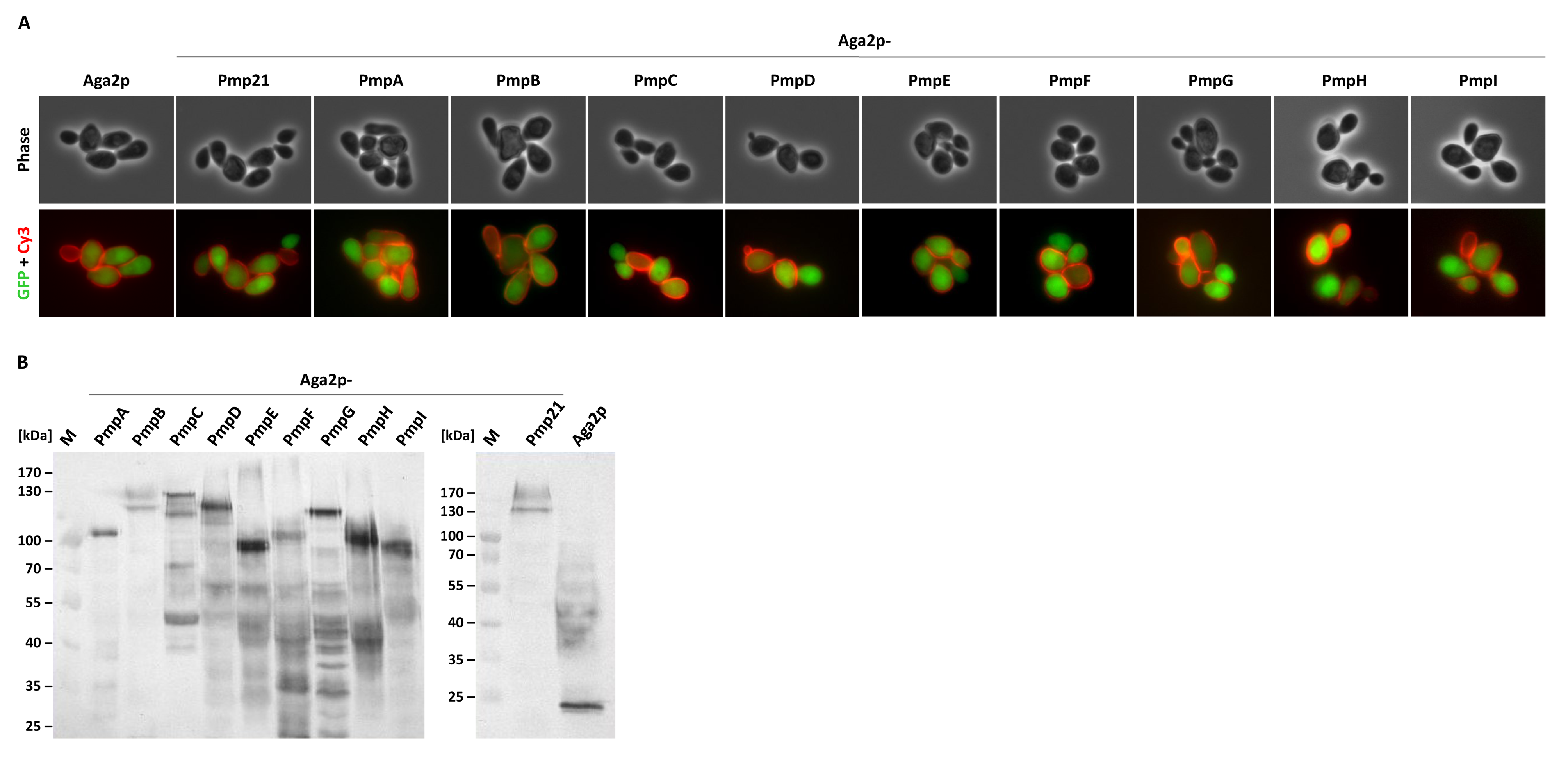

Supplement: Figure S1 — Yeast cells presenting chlamydial Pmps on their cell surface. (A) Detection of Aga2 and Aga2-Pmp fusion proteins on the yeast cell surface. Green fluorescent yeast cells expressing Aga2 and Aga2–Pmp fusion proteins were fixed and stained with an anti-V5 antibody (red) against the C-terminal V5-Tag. Top panel: Phase contrast microscopy. Bottom panel: Fluorescence microscopy. (B) Western blot analysis of Aga2 and Aga2-Pmp fusion proteins. Total protein extracts from yeast strains expressing Aga2 or Aga2–Pmp fusion proteins were digested with α-mannosidase to remove Aga2 O-glycosylation, resolved by SDS-PAGE and probed with an anti-His antibody. Protein size markers (in kDa) are indicated to the left of each blot. [file mbo30003-0544-sd1.tif]

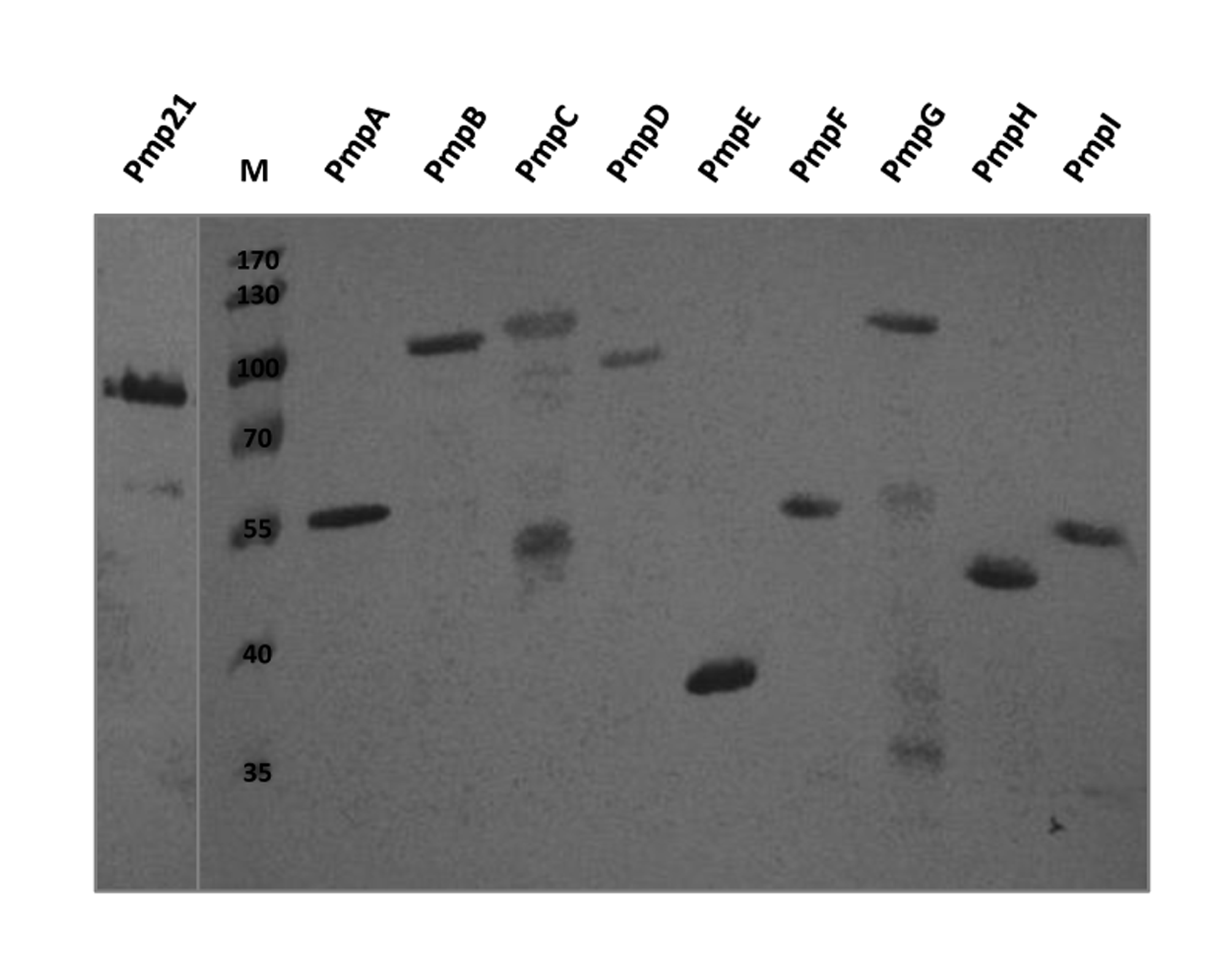

Supplement: Figure S2 — Coating efficiency of latex beads. Western blot analysis of recombinant proteins coated on 2 × 10^7^ latex beads (adhesion data shown in Fig. C). Proteins were removed from the bead surface by SDS-loading buffer and DTT, resolved by SDS-PAGE, and probed with an antibody against the N-terminal His tag. Lane M, molecular mass marker. [file mbo30003-0544-sd2.tif]
