# Supplementary material for: All subtypes of the Pmp adhesin family are implicated in chlamydial virulence and show species-specific function
Source: Microbiologyopen. 2014 Jul 1;3(4):544–56. doi: 10.1002/mbo3.186 (PMC4287181; doi:10.1002/mbo3.186)
Supplement: Table S1 — List of oligonucleotides used for plasmid construction. [file mbo30003-0544-sd3.pdf]

Table S1: List of oligonucleotides used for plasmid construction.

Gene	Vector	Primer	Primer sequence (5' to 3')
PmpA	pYD1	C-1932	CGACGATGACGACAAGGTACCAGGATCCAGTGTGGTGGAA **GGATCTGAAAGCACAGGAGG**
		C-1933	TAGGGATAGGCTTACCTTCGAAGGGCCCTCTAGACTCGAG **GGTGAGCAAGATTTCCATTT**
	pKM32	C-2035	CCATCACCATCACCATACGGATCCGCATGCGAGCTCGGTA **GGATCTGAAAGCACAGGAGG**
		C-2036	CAGGAGTCCAAGCTCAGCTAATTAAGCTTGGCTGCAGGTC **GGTGAGCAAGATTTCCATTT**
PmpB	pYD1	C-1934	CGACGATGACGACAAGGTACCAGGATCCAGTGTGGTGGAA **GAAGGCGGTGCTATCTTCTC**
		C-1935	TAGGGATAGGCTTACCTTCGAAGGGCCCTCTAGACTCGAG **AGGTGCTGAAGCTGTTGTTG**
	pKM32	C-2037	CCATCACCATCACCATACGGATCCGCATGCGAGCTCGGTA **GAAGGCGGTGCTATCTTCTC**
		C-2040	CAGGAGTCCAAGCTCAGCTAATTAAGCTTGGCTGCAGGTC **AGGTGCTGAAGCTGTTGTTG**
PmpC	pYD1	C-1936	CGACGATGACGACAAGGTACCAGGATCCAGTGTGGTGGAA **AACGGAGGAGCTATTTATGG**
		C-1937	TAGGGATAGGCTTACCTTCGAAGGGCCCTCTAGACTCGAG **GACACTCTCGTCAGTTATTT**
	pKM32	C-2041	CCATCACCATCACCATACGGATCCGCATGCGAGCTCGGTA **AACGGAGGAGCTATTTATGG**
		C-2042	CAGGAGTCCAAGCTCAGCTAATTAAGCTTGGCTGCAGGTC **GACACTCTCGTCAGTTATTT**
PmpD	pYD1	C-1938	CGACGATGACGACAAGGTACCAGGATCCAGTGTGGTGGAA **TCTTTTGAAGGAAACAGCGC**
		C-1939	TAGGGATAGGCTTACCTTCGAAGGGCCCTCTAGACTCGAG **AGAATTTCCTTTAAAAACAA**
	pKM32	C-1881	CCATCACCATCACCATACGGATCCGCATGCGAGCTCGGTA **TCTTTTGAAGGAAACAGCGC**
		C-1882	GGAGTCCAAGCTCAGCTAATTAAGCTTGGCTGCAGGTC **AGAATTTCCTTTAAAAACAATA**
PmpE	pYD1	C-1940	CGACGATGACGACAAGGTACCAGGATCCAGTGTGGTGGAA **GATACACAAAAAGAAGGTAT**
		C-1941	TAGGGATAGGCTTACCTTCGAAGGGCCCTCTAGACTCGAG **TCTCCTAGGAGGATTAGTAC**
	pKM32	C-2043	CCATCACCATCACCATACGGATCCGCATGCGAGCTCGGTA **GATACACAAAAAGAAGGTAT**
		C-2044	CAGGAGTCCAAGCTCAGCTAATTAAGCTTGGCTGCAGGTC **TCTCCTAGGAGGATTAGTAC**

PmpF	pYD1	C-1942	CGACGATGACGACAAGGTACCAGGATCCAGTGTGGTGGAA **CTCTTCTGCAACAACTACTG**
		C-1943	TAGGGATAGGCTTACCTTCGAAGGGCCCTCTAGACTCGAG **CGGATTTTGGGTAAACGGGA**
	pKM32	C-2045	CCATCACCATCACCATACGGATCCGCATGCGAGCTCGGTA **CTCTTCTGCAACAACTACTG**
		C-2046	CAGGAGTCCAAGCTCAGCTAATTAAGCTTGGCTGCAGGTC **CGGATTTTGGGTAAACGGGA**
PmpG	pYD1	C-1944	CGACGATGACGACAAGGTACCAGGATCCAGTGTGGTGGAA **GAAATCATGGTTCCTCAAGG**
		C-1945	TAGGGATAGGCTTACCTTCGAAGGGCCCTCTAGACTCGAG **ATCCACACTTGCTTGAATTG**
	pKM32	C-1751	CCATCACCATCACCATACGGATCCGCATGCGAGCTCGGTA **GCAGAAATCATGATTCCTCA**
		C-1752	CAGGAGTCCAAGCTCAGCTAATTAAGCTTGGCTGCAGGTC **ATCCACACTTGCTTGAATTG**
PmpH	pYD1	C-1946	CGACGATGACGACAAGGTACCAGGATCCAGTGTGGTGGAA **ATGTTCTCGAAAAATGTTTC**
		C-1947	TAGGGATAGGCTTACCTTCGAAGGGCCCTCTAGACTCGAG **GCTGCGCTCTCCAGGACGAT**
	pKM32	C-2047	CCATCACCATCACCATACGGATCCGCATGCGAGCTCGGTA **ATGTTCTCGAAAAATGTTTC**
		C-2048	CAGGAGTCCAAGCTCAGCTAATTAAGCTTGGCTGCAGGTC **GCTGCGCTCTCCAGGACGAT**
PmpI	pYD1	C-1948	CGACGATGACGACAAGGTACCAGGATCCAGTGTGGTGGAA **AGTTTCTGTCGAAATCATGC**
		C-1949	TAGGGATAGGCTTACCTTCGAAGGGCCCTCTAGACTCGAG **GAGAGGAATATTGTTTTGCT**
	pKM32	C-2049	CCATCACCATCACCATACGGATCCGCATGCGAGCTCGGTA **AGTTTCTGTCGAAATCATGC**
		C-2050	CAGGAGTCCAAGCTCAGCTAATTAAGCTTGGCTGCAGGTC **GAGAGGAATATTGTTTTGCT**
Cpn0498	pFT8	C-1156	AGGTCGTCTGGAAGTTCTGTTCCAGGGGCCCCTGGGATCCA **AATCAAGATGTTCTATTGATAA**
		C-1157	GAGGCAGATCGTCAGTCAGTCAATGGTGATGGTGATGGTG **TCCAAAACAACCCTCTGAAATA**

Bold nucleotides are homologous to sequences within the gene indicated.
